# Hazard Ratio Analysis of Laparoscopic Radical Hysterectomy for IA1 With LVSI-IIA2 Cervical Cancer: Identifying the Possible Contraindications of Laparoscopic Surgery for Cervical Cancer

**DOI:** 10.3389/fonc.2020.01002

**Published:** 2020-07-08

**Authors:** Pengfei Li, Ping Liu, Ying Yang, Lu Wang, Jiaqi Liu, Xiaonong Bin, Jinghe Lang, Chunlin Chen

**Affiliations:** ^1^Department of Obstetrics and Gynecology, Nanfang Hospital, Southern Medical University, Guangzhou, China; ^2^Department of Obstetrics and Gynecology, Xinqiao Hospital, Army Medical University, Chongqing, China; ^3^Department of Epidemiology, College of Public Health, Guangzhou Medical University, Guangzhou, China; ^4^Department of Obstetrics and Gynecology, Peking Union Medical College Hospital, Peking Union Medical College, Beijing, China

**Keywords:** cervical cancer, radical hysterectomy, laparoscopy, oncological outcomes, hazard ratio

## Abstract

**Objectives:** This study aimed to compare the 5-year disease-free survival (DFS) and overall survival (OS) of laparoscopic radical hysterectomy (LRH) and abdominal radical hysterectomy (ARH) for IA1 with lymphovascular space invasion (LVSI)-IIA2 cervical cancer and to analyze the Cox proportional hazard ratio (HR) of LRH among the total study population and different subgroups.

**Methods:** This was a multicenter retrospective cohort study. The oncological outcomes of LRH (*n* = 4,236) and ARH (*n* = 9,177) were compared. The HRs and 95% confidence intervals for the effect of LRH on 5-year OS and DFS were estimated by Cox proportional hazards models.

**Results:** Overall, there was no difference in DFS between LRH and ARH in the unadjusted analysis (HR 1.11, 95% CI: 0.99–1.25, *p* = 0.075). The risk-adjusted analysis revealed that LRH was independently associated with inferior DFS (HR 1.25, 95% CI: 1.11–1.40, *p* < 0.001). There was no difference in OS between the two groups in the unadjusted analysis (HR 1.00, 95% CI: 0.85–1.17, *p* = 0.997) or risk-adjusted analysis (HR 1.15, 95% CI: 0.98–1.35, *p* = 0.091). For patients with FIGO stage IB1 and tumor size <2 cm, LRH was not associated with lower DFS or OS (*p* = 0.637 or *p* = 0.107, respectively) in risk-adjusted analysis. For patients with FIGO stage IB1 and tumor size ≥2 cm, LRH was associated with lower 5-year DFS (HR 1.42, 95% CI: 1.19–1.69, *p* < 0.001) in risk-adjusted analysis, but it was not associated with lower 5-year OS (*p* = 0.107). For patients with FIGO stage IIA1 and tumor size <2 cm, LRH was not associated with lower 5-year DFS or OS (*p* = 0.954 or *p* = 0.873, respectively) in risk-adjusted analysis. For patients with FIGO stage IIA1 and tumor size ≥2 cm, LRH was associated with lower DFS (HR 1.48, 95% CI: 1.16–1.90, *p* = 0.002) and 5-year OS (HR 1.69, 95% CI: 1.22–2.33, *p* = 0.002) in risk-adjusted analysis.

**Conclusion:** The 5-year DFS of LRH was worse than that of ARH for FIGO stage IA1 with LVSI-IIA2. LRH is not an appropriate option for FIGO stage IB1 or IIA1 and tumor size ≥ 2 cm compared with ARH.

## Introduction

Cervical cancer is the fourth most common cancer among women worldwide; 85% of new cases and 90% of deaths are from low-resource regions or from people who live in socioeconomically weaker sections of society, and the disease seriously threatens women's health (1–3). China faces a heavy burden of cervical cancer, with 98,900 new cases and 30,500 deaths annually (4). Primary surgery consisting of radical hysterectomy plus pelvic lymphadenectomy is the most appropriate option for patients with stage IB1 or IIA1 disease and an alternative to concomitant chemoradiation therapy for patients with stage IB2 or IIA2 disease (3, 5). Select stage IIA2 patients may also be treated with surgery in certain Asian and European countries (6, 7).

Initially, data suggested comparable oncological outcomes from minimally invasive radical hysterectomy (robotic or laparoscopic) compared to abdominal radical hysterectomy (ARH) (8–10). Similarly, laparoscopic radical hysterectomy (LRH) appears to provide equivalent or better intraoperative and short-term postoperative outcomes (11). These findings have led to widespread use of LRH for cervical cancer. However, a phase III randomized clinical trial demonstrated that minimally invasive radical hysterectomy was associated with lower rates of 4.5-year disease-free survival (DFS) and overall survival (OS) compared with ARH (12). Several recent retrospective studies also demonstrated that minimally invasive radical hysterectomy was associated with shorter survival compared with ARH (13–15). Based on this evidence, an open abdominal approach is recommended as the only standard approach for radical hysterectomy starting with Cervical Cancer, Version 3.2019, NCCN Clinical Practice Guidelines in Oncology (16).

Because of the safety of laparoscopic surgery in endometrial cancer (17), prostate cancer (18), gastric cancer (19), and other abdominal or pelvic malignancies, the results that LRH was associated with worse oncological outcomes were unexpected (20). Current studies have failed to explain the reasons for these results. In some studies, it has also been suggested that LRH is as effective as ARH among patients with stage IB1 cervical cancer and those with a cervical mass size of ≤2 cm (15, 21). Therefore, we hypothesized that laparoscopic surgery, as a widely accepted minimally invasive method, is effective in specific subgroups of patients with cervical cancer and ineffective in other subgroups; that is, there are indications and contraindications for LRH. However, there are few correlative studies.

Thus, the main objective of the current study was to analyze the hazard ratios (HRs) of LRH for stage IA1 with lymphovascular space invasion (LVSI)-IIA2 cervical cancer compared with those of ARH and to identify the subgroups in which LRH was associated with statistically shorter survival compared with ARH, which may be helpful in identifying possible contraindications of LRH in the treatment of cervical cancer.

## Methods

### Data Source

This study was a multicenter, retrospective, cohort study, and the data of this study originated from the clinical diagnosis and treatment for cervical cancer in mainland China (Four C) database, a cervical cancer-specialized disease database (*n* = 46,313) that covers consecutive patients with cervical cancer in 37 hospitals in mainland China treated between January 2004 and December 2016. This study was carried out in accordance with the ethical standards of the institutional and/or national research committee. The Ethics Committee of Nanfang Hospital, Southern Medical University, approved this retrospective study (ethics number NFEC-2017-135). The written informed consent was waived by the ethics committee, since the information of human's medical documents was retrospectively gathered and analyzed and human data were unidentifiable in this study. The identifier of the clinical trial is CHiCTR180017778 (International Clinical Trials Registry Platform Search Port, http://apps.who.int/trialsearch/).

Clinical data were collected from patient files and the medical record management system in the hospitals by trained gynecological oncology staff using standardized data collection and quality control procedures. The details of the data sources and methods were the same as previously reported (22, 23). For patients undergoing surgical treatment, the collected data, including the demographic details, preoperative examination results, surgical information, pathological results, preoperative, and postoperative adjuvant treatment details, complications, hospitalization time, and expenses, and follow-up, contained almost all the information during the treatment of cervical cancer. In this database, the FIGO stage was recorded and corrected by tumor size according to the FIGO 2009 staging system. Tumor size was evaluated by pathological records. To ensure the accuracy of the collected data, two uniformly trained staff used EpiData software (EpiData Association, Odense M, Denmark) to input and proofread the same data from each hospital.

All follow-up procedures were carried out by trained gynecological oncology staff at each center to keep the patients' personal data confidential and to provide disease management guidance at the same time. The follow-up information was gathered through the return visit system or telephone follow-up, including survival status, time of death, recurrence time, recurrence site, and treatment after recurrence. The oncological outcomes were estimated according to the recorded information, and the last day of the return visit or telephone follow-up was defined as the last follow-up. The follow-up rate of oncological outcomes was 72.7% in this database.

### Cohort Selection

**Figure 1:**
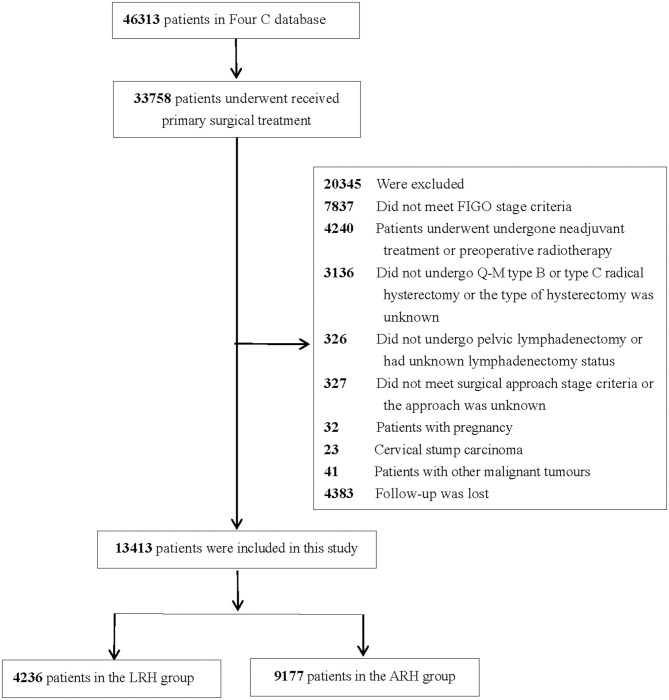
Flowchart of patients included in the analysis.

The inclusion criteria were as follows: received primary surgical treatment, met FIGO stage IA1 with LVSI-IIA2, underwent upfront radical hysterectomy + pelvic lymphadenectomy ± para-abdominal aortic lymphadenectomy, and had total laparoscopic or laparotomy as a surgical approach. The exclusion criteria were as follows: patients lost to follow up, patients who underwent laparoscopically assisted vaginal surgery, patients with pregnancy, patients with cervical stump carcinoma, patients with other malignant tumors, and patients who did not meet the inclusion criteria. The flow diagram of recruitment and exclusion is shown in Figure 1.

### Definitions and Outcome Measures

The OS rate and DFS rate of LRH and ARH were calculated by observing the long-term oncology outcomes within 5 years after surgery. OS was defined as the time from the date of surgery to the date of death from any cause. DFS was defined as the time from the date of surgery to the date of disease recurrence or death from cervical cancer, and patients with no evidence of recurrence or death were censored at the date of the last follow-up or return visit. Superficial stromal invasion was defined as the depth of stromal invasion at <10 mm or <1/2 of the stromal depth, and deep stromal invasion was defined as the depth of stromal invasion at ≥10 mm or ≥1/2 of the stromal depth.

### Statistical Analysis

All statistical procedures were processed with SPSS 23.0 statistical software (SPSS, Inc., Chicago, IL, USA). The between-group differences in the baseline characters were assessed through independent two-sample *t* tests or Pearson's chi-squared test. The quantitative data are shown as the mean ± standard deviation (*x* ± *s*), and the nominal-scale data are shown as percentages (%). The 5-year OS and DFS rates of LRH and ARH were calculated and compared using the Kaplan–Meier curve and the log-rank test. A Cox proportional hazards model was used to estimate the HRs and 95% confidence intervals for the effect of treatment on 5-year OS and DFS; the known factors that may affect the oncological outcome of cervical cancer were included in this multivariate model to adjust for case mix, including age, FIGO stage, operative approach, tumor size (<2 vs. ≥2 cm), parametrial tumor involvement, stromal invasion, LVSI, lymph node metastasis, surgical margin invasion, and postoperative adjuvant treatment. A *p* values <0.05 from two-sided tests was regarded as significant.

## Results

Among the 46,313 patients described in the database, 13,413 were included in this study (4,236 patients in the LRH group and 9,177 patients in the ARH group). The clinicopathologic characteristics of the two groups are shown in Table 1. The mean age between the LRH group and ARH group was statistically different (*p* < 0.001). Patients in the LRH group were more likely to have lower stage disease than those in the ARH group (*p* < 0.001). Histology in the LRH group was less likely to be squamous cell and more likely to be adenocarcinoma than that in the ARH group (*p* < 0.001). Patients in the LRH group were more likely to have LVSI than those in the ARH group (*p* = 0.008), and patients in the ARH group were more likely to have lymph node metastasis, positive surgical margins, tumor size ≥2 cm, and deep stromal invasion than those in the LRH group (all *p* < 0.05).

**Table 1:** Clinicopathologic characteristics of patients in the ARH and LRH groups.

**Characteristic**	**ARH**	**LRH**	***p***
	***n* = 9,177 (%)**	***n* = 4,236 (%)**	
**Age (years)**	48.08 ± 9.706	47.43 ± 9.288	<0.001
**FIGO stage**			<0.001
IA	191 (2.1)	118 (2.8)	
IB1	5,557 (60.6)	2,929 (69.1)	
IB2	716 (7.8)	299 (7.1)	
IIA1	2,263 (24.7)	749 (17.7)	
IIA2	450 (4.9)	141 (3.3)	
**Histology**			<0.001
Squamous cell carcinoma	8,008 (87.3)	3,535 (83.5)	
Adenocarcinoma	720 (7.8)	487 (11.5)	
Adenosquamous	226 (2.5)	81 (1.9)	
Uncommon pathological type	176 (1.9)	84 (2)	
Unknown	47 (0.5)	49 (1.2)	
**Tumor size**			<0.001
<2 cm	1,185 (12.9)	764 (18)	
≥2 cm	7,419 (80.8)	3,011 (71.1)	
Unknown	573 (6.2)	461 (10.9)	
**Stromal invasion**			<0.001
Superficial	3,631 (39.6)	1,944 (45.9)	
Deep	4,944 (53.9)	1,850 (43.7)	
Unknown	602 (6.6)	442 (10.4)	
**Lymphovascular space invasion**			0.008
Yes	1,731 (18.9)	882 (20.8)	
No	7,446 (81.1)	3,354 (79.2)	
**Parametrial tumor involvement**			0.556
Yes	160 (1.7)	80 (1.9)	
No	9,017 (98.3)	4,156 (98.1)	
**Surgical margin invasion**			0.002
Yes	212 (2.3)	64 (1.5)	
No	8,965 (97.7)	4,172 (98.5)	
**Lymph node metastasis**			0.001
Yes	1,645 (17.9)	662 (15.6)	
No	7,532 (82.1)	3,574 (84.4)	
**Adjuvant therapy**			<0.001
None	3,106 (33.8)	1,724 (40.7)	
Chemotherapy	1,493 (16.3)	1,054 (24.9)	
Radiotherapy/radiochemotherapy	4,578 (49.9)	1,458 (34.4)	

**Figure 2:**
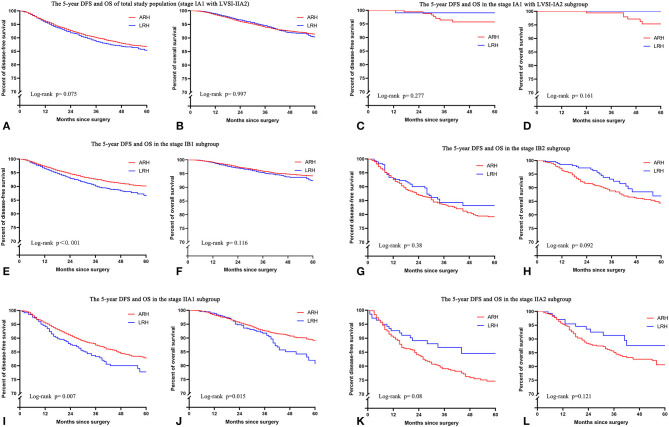
The unadjusted Kaplan-Meier survival curves of the entire cohort and different stages. **(A,B)** Stage IA1 with LVSI-IIA2, **(C,D)** stage IA1 with LVSI-IA2, **(E,F)** stage IB1, **(G,H)** stage IB2, **(I,J)** stage IIA1, **(K,L)** stage IIA2.

In the total study population, the unadjusted 5-year DFS rate of LRH and ARH for stage IA1 with LVSI-IIA2 cervical cancer was similar (85.1 vs. 86.6%; HR 1.11, 95% CI: 0.99–1.25, *p* = 0.075). And the unadjusted 5-year OS rate of LRH and ARH was also similar (90.4 vs. 91.4%; HR 1.00, 95% CI: 0.85–1.17, *p* = 0.997). The unadjusted Kaplan–Meier survival curves are shown in Figures 2A,B. After adjusting for case mix with multivariable analysis, LRH was associated with a shorter 5-year DFS (HR 1.25, 95% CI: 1.11–1.40, *p* < 0.001), but it was not associated with a shorter 5-year OS (HR 1.15, 95% CI: 0.98–1.35, *p* = 0.091).

In the FIGO stage IB1 subgroup, patients who underwent LRH showed decreased 5-year DFS rates (86.5 vs. 90.0%; HR 1.32, 95% CI: 1.13–1.54, *p* < 0.001) and similar 5-year OS rates (92.4 vs. 94.1%; HR 1.19, 95% CI: 0.96–1.48, *p* = 0.116) compared to those who underwent ARH in the unadjusted analysis (Figures 2E,F and Table 2). The risk-adjusted analysis revealed that LRH was associated with a lower 5-year DFS rate (HR 1.39, 95% CI: 1.18–1.62, *p* < 0.001), but it was not associated with a lower 5-year OS rate (HR 1.24, 95% CI: 0.99–1.55, *p* = 0.058).

**Table 2:** Unadjusted and adjusted hazard ratios of LRH compared with ARH.

**Study population**	**Unadjusted 5-year DFS**		**Adjusted 5-year DFS**		**Unadjusted 5-year OS**		**Adjusted 5-year OS**	
	**HR (95% CI)**	***p***	**HR (95% CI)**	***p***	**HR (95% CI)**	***p***	**HR (95% CI)**	***p***
Total study population	1.11 (0.99–1.25)	0.075	1.25 (1.11–1.40)	<0.001	1.00 (0.85–1.17)	0.997	1.15 (0.98–1.35)	0.091
Stage IA1 with LVSI to IA2	0.33 (0.04–2.70)	0.301	0.41 (0.05–3.49)	0.415	0.03 (0–88.08)	0.394	0.001 (0–4.5E+14)	0.722
Stage IB1	1.32 (1.13–1.54)	<0.001	1.39 (1.18–1.62)	<0.001	1.19 (0.96–1.48)	0.116	1.24 (0.99–1.55)	0.058
Stage IB2	0.85 (0.60–1.21)	0.382	0.81 (0.56–1.17)	0.249	0.66 (0.41–1.07)	0.094	0.67 (0.41–1.10)	0.11
Stage IIA1	1.36 (1.09–1.70)	0.007	1.40 (1.11–1.77)	0.005	1.44 (1.07–1.94)	0.015	1.52 (1.12–2.07)	0.008
Stage IIA2	0.64 (0.39–1.06)	0.083	0.72 (0.43–1.19)	0.199	0.62 (0.34–1.14)	0.126	0.69 (0.37–1.29)	0.243
Stage IB1 and tumor size <2 cm	1.16 (0.72–1.85)	0.546	1.12 (0.70–1.81)	0.637	1.79 (0.87–3.70)	0.115	1.84 (0.88–3.84)	0.107
Stage IB1 and tumor size ≥ 2 cm	1.43 (1.21–1.70)	<0.001	1.42 (1.19–1.69)	<0.001	1.27 (1.01–1.62)	0.045	1.22 (0.96–1.55)	0.107
Stage IIA1 and tumor size <2 cm	0.94 (0.39–2.27)	0.889	1.03 (0.41–2.56)	0.954	0.85 (0.23–3.17)	0.805	1.12 (0.27–4.69)	0.873
Stage IIA1 and tumor size ≥ 2 cm	1.41 (1.11–1.80)	0.005	1.48 (1.16–1.90)	0.002	1.57 (1.15–2.14)	0.004	1.69 (1.22–2.33)	0.002

In the FIGO stage IIA1 subgroup, patients who underwent LRH had decreased 5-year DFS (77.7 vs. 82.6%; HR 1.36, 95% CI: 1.09–1.70, *p* = 0.007) and OS (80.6 vs. 89.0%; HR 1.44, 95% CI: 1.07–1.94, *p* = 0.015) rates compared to those who underwent ARH in the unadjusted analysis (Figures 2I,J and Table 2), and LRH was associated with worse 5-year DFS (HR 1.40, 95% CI: 1.11–1.77, *p* = 0.005) and OS (HR 1.52, 95% CI: 1.12–2.07, textitp = 0.008) rates in the risk-adjusted analysis.

In the subgroups of FIGO stage IA1 with LVSI to IA2, IB2, and IIA2, patients who underwent LRH showed similar 5-year DFS and OS rates in the unadjusted or risk-adjusted analyses (all *p* > 0.05), as shown in Figures 2C,D,G,H,K,L and Table 2.

**Figure 3:**
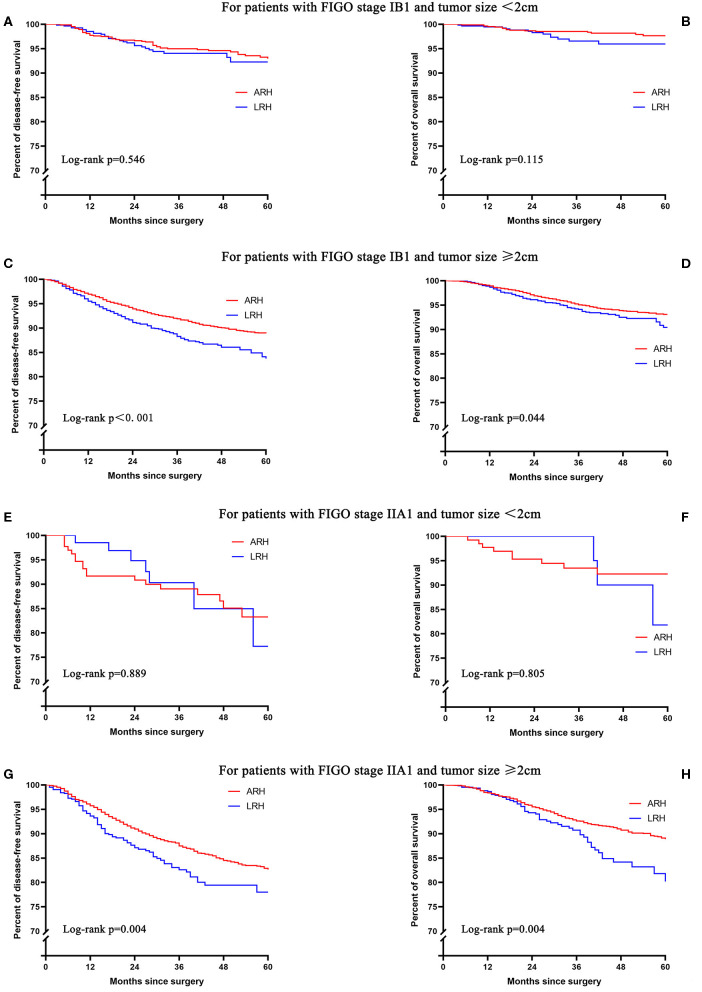
The unadjusted Kaplan-Meier survival curves of stage IB1 or IIA1 with different tumor sizes. **(A,B)** stage IB1 and tumor size <2 cm, **(C,D)** stage IB1 and tumor size ≥2 cm, **(E,F)** stage IIA1 and tumor size <2 cm, **(G,H)** stage IIA1 and tumor size ≥2 cm.

For patients with FIGO stage IB1 or IIA1 and tumor size <2 cm, the 5-year DFS and OS rates of LRH and ARH were similar in the unadjusted analysis (Figures 3A,B,E,F and Table 2). The risk-adjusted analysis revealed that LRH was not identified as an independent risk factor for worse 5-year DFS or OS (all *p* > 0.05), as shown in Table 2.

For patients with FIGO stage IB1 and tumor size ≥2 cm, the 5-year DFS (83.7 vs. 88.8%; HR 1.43, 95% CI: 1.21–1.70, *p* < 0.001) and OS (90.4 vs. 93.1%; HR 1.27, 95% CI: 1.01–1.62, *p* = 0.045) rates of LRH were significantly lower than those of ARH in the unadjusted analysis (Figures 3C,D and Table 2). The risk-adjusted analysis revealed that LRH was associated with lower 5-year DFS (HR 1.42, 95% CI: 1.19–1.69, *p* < 0.001), but it was not associated with lower 5-year OS (HR 1.22, 95% CI: 0.96–1.55, *p* = 0.107).

For patients with FIGO stage IIA1 and tumor size ≥2 cm, the 5-year DFS (77.9 vs. 82.6%; HR 1.41, 95% CI: 1.11–1.80, *p* = 0.005) and OS (80.2 vs. 88.9%; HR 1.57, 95% CI: 1.15–2.14, *p* = 0.004) rates of LRH were significantly lower than those of ARH in the unadjusted analysis (Figures 3G,H and Table 2). The risk-adjusted analysis revealed that LRH was associated with lower DFS (HR 1.48, 95% CI: 1.16–1.90, *p* = 0.002) and 5-year OS (HR 1.69, 95% CI: 1.22–2.33, *p* = 0.002).

## Discussion

In this study, we compared the oncological outcomes of LRH and ARH for IA1 with LVSI-IIA2 cervical cancer. We found that LRH showed similar 5-year DFS and OS rates to ARH in the unadjusted analysis, which may explain why the conclusion that LRH was associated with worse oncological outcomes among cervical cancer patients was unexpected. The underlying reason is that clinicians tend to choose patients with more advanced-stage disease and with more high-risk or intermediate-risk factors to undergo ARH instead of LRH. This fact gives most clinicians the misunderstanding that the long-term survival outcomes of LRH are comparable to those of ARH. After adjusting for case mix with the multivariable analysis, we found that LRH was associated with a shorter 5-year DFS but was not associated with a shorter 5-year OS. This result is consistent with the results of several recent studies on the survival outcomes of LRH in cervical cancer patients (15).

We also conducted analysis in the subgroups of different FIGO stages and found that LRH was identified as an independent risk factor for worse 5-year DFS or OS in the FIGO stage IB1 and IIA1 subgroups. Then we performed stratified analysis for different tumor sizes among patients with FIGO stage IB1 or IIA1. We found that LRH was not identified as an independent risk factor for worse 5-year DFS or OS in the subgroups of FIGO stage IB1 or IIA1 and tumor size <2 cm either in the unadjusted analysis or in the risk-adjusted analysis; this finding suggests that LRH may be suitable for patients with FIGO stage IB1 or IIA1 and tumor size <2 cm. LRH was identified as an independent risk factor for worse 5-year DFS or OS in the subgroups of FIGO stage IB1 or IIA1 and tumor size ≥2 cm; this finding suggests that LRH should be carefully considered for patients with FIGO stage IB1 or IIA1 and tumor size ≥2 cm.

This large sample retrospective cohort study adds to the evidence that LRH may be a prognostic factor for cervical cancer, and the results of subgroup analysis suggest that LRH may be suitable for selected patients. LRH was found not to be associated with worse 5-year DFS or OS for patients with FIGO stage IB1 or IIA1 disease and tumor size <2 cm, which was consistent with the results of several recently published studies (13–15). There are also two studies with different results. Paik et al. (24) found that LRH was associated with a lower rate of DFS among patients with IB–IIA and tumor size <2 cm (ARH 186 vs. LRH 62). Uppal et al. (25) found that minimally invasive surgery was associated with a higher likelihood of recurrence in the risk-adjusted analysis of IA1 with LVSI to IB1 patients with tumor size ≤2 cm on final pathology (ARH 82 vs. minimally invasive surgery 182). In Paik's study, tumor size was determined by clinical palpation or inspection, but tumor size classification on clinical evaluation seemed to be different from the tumor size classification on final pathology, so some patients with tumor size >2 cm on final pathology may be included in the analysis. A larger percentage of minimally invasive surgery was robotic-assisted surgery in Uppal's study, while all of that was laparoscopic surgery in our study, which is a possible cause of the different results between the two studies.

Patients with IB2 and IIA2 cervical cancer were included because there is still a considerable number of stage IB2 and IIA2 patients in mainland China who choose surgery as the primary treatment (7), which may be due to the high costs of radiotherapy and the relative lack of radiotherapy devices. Although radiotherapy devices in mainland China have increased significantly in the past three decades, the number of accelerators per million people in mainland China in 2015 was only 1.42, which was much lower than two to three accelerators per million people recommended by the World Health Organization (26). Regional imbalance and lack of afterloading radiotherapy equipment also affected the choice of treatment for patients with IB2 and IIA2 cervical cancer. The availability of radiotherapy resources affects the choice of primary treatment, and patients with advanced disease may be more likely to receive surgical treatment in low-resource regions.

Patients with one or more high-risk factors (lymph node metastasis, parametrial tumor involvement, and surgical margin invasion) were recommended to receive postoperative adjuvant chemoradiation therapy. Patients with two or more intermediate-risk factors (deep cervical stromal invasion, tumor size >4 cm, and LVSI) were recommended to receive postoperative adjuvant radiation or chemoradiation therapy. But in real clinical practice, there are still a small number of patients receiving chemotherapy alone. To minimize the impact of postoperative adjuvant treatment on the results of this study, we included them as an influencing variable in the multivariate analysis.

Our study had some limitations. First, the patient files and medical records may be different among hospitals, leading to a lack of certain clinical data. Second, although the study included cervical cancer patients from 37 hospitals, it did not completely cover all institutions in mainland China. Third, patients with IB2 and IIA2 cervical cancer who received surgical treatment were included in this study, although the majority of patients with IB2 and IIA2 diseases in other countries were treated with definitive chemoradiation; this may make the conclusions drawn not broadly applicable, and we conducted subgroups analysis among patients with FIGO stage IB1 and IIA1 diseases to eliminate this limitation. Fourth, this study was a retrospective study, and the oncological outcomes of 27.3% of patients in the database are unknown, so the findings are subject to the biases and confounding factors.

## Conclusions

Patients with advanced-stage disease and with more high-risk or intermediate-risk factors are likely to undergo ARH instead of LRH, causing a false impression that the oncological outcomes of LRH and ARH for cervical cancer patients are comparable. After adjusting for case mix with the multivariable analysis, the 5-year DFS rate of LRH was worse than that of ARH for FIGO stage IA1 with LVSI-IIA2. Compared with ARH, LRH is not an appropriate option for FIGO stage IB1 or IIA1 and tumor size ≥2 cm. More studies are needed to clarify the indications and contraindications for LRH.

## Data Availability Statement

The datasets generated for this study are available on request to the corresponding author.

## Ethics Statement

The establishment of the cervical cancer database was reviewed and approved by the ethics committee of Nanfang Hospital, Southern Medical University (ethics number NFEC-2017-135). Written informed consent was not provided because this study is a retrospective study, in which only the information of patient's medical documents were was gathered and analyzed, and human data was unidentifiable in this study, the ethics committee exempted informed consent.

## Author Contributions

CC, YY, and JLa: conceptualization. JLi, PLi, and LW: data curation. PLi, LW, and JLi: writing—original draft. CC, PLi, and XB: statistical analysis. CC: funding acquisition. CC and PLi: project administration. CC, PLiu, and JLa: supervision. All authors: writing—review and editing.

## Conflict of Interest

The authors declare that the research was conducted in the absence of any commercial or financial relationships that could be construed as a potential conflict of interest.
